# Impact of a Faulty Germinal Center Reaction on the Pathogenesis of Primary Diffuse Large B Cell Lymphoma of the Central Nervous System

**DOI:** 10.3390/cancers13246334

**Published:** 2021-12-17

**Authors:** Manuel Montesinos-Rongen, Anna Brunn, Monica Sanchez-Ruiz, Ralf Küppers, Reiner Siebert, Martina Deckert

**Affiliations:** 1 Institute of Neuropathology, Faculty of Medicine, University Hospital Cologne, 50937 Cologne, Germany; manuel.montesinos-rongen@uk-koeln.de (M.M.-R.); anna.brunn@uk-koeln.de (A.B.); monica.sanchez-ruiz@uk-koeln.de (M.S.-R.); 2 Institute of Cell Biology (Cancer Research), Medical School, University of Duisburg-Essen, 45122 Essen, Germany; Ralf.Kueppers@uk-essen.de; 3 Institute of Human Genetics, Ulm University and Ulm University Medical Center, 89081 Ulm, Germany; reiner.siebert@uni-ulm.de

**Keywords:** PCNSL, diffuse large B cell lymphoma, B cell receptor, germinal center, somatic hypermutation, CNS, animal model, mouse, microenvironment, molecular pathogenesis

## Abstract

**Simple Summary:**

The pathogenetic mechanisms and peculiar tropism of primary CNS lymphoma (PCNSL) of the central nervous system (CNS) have been the subject of debate for decades. Hypothesis-driven targeted molecular studies have revealed that PCNSLs derived from self-/polyreactive B cells that have escaped developmental control mechanisms. The early acquisition of activating mutations targeting the B cell receptor pathway provides a survival advantage. The failure of the germinal center (GC) reaction and its checkpoints increases tumor B cell affinity for the CNS. During this faulty GC reaction, PCNSL tumor cells acquire further oncogenic alterations converging on the Toll-like receptor, B cell receptor, and NF-κB pathway. These activated pathways sustain proliferation. Concomitantly, cells become unable to complete terminal B cell differentiation, becoming trapped within the vicious cycle of the GC reaction as low-affinity IgM+ B cells related to memory cells.

**Abstract:**

Primary lymphoma of the central nervous system (PCNSL, CNS) is a specific diffuse large B cell lymphoma (DLBCL) entity confined to the CNS. Key to its pathogenesis is a failure of B cell differentiation and a lack of appropriate control at differentiation stages before entrance and within the germinal center (GC). Self-/polyreactive B cells rescued from apoptosis by *MYD88* and/or *CD79B* mutations accumulate a high load of somatic mutations in their rearranged immunoglobulin (IG) genes, with ongoing somatic hypermutation (SHM). Furthermore, the targeting of oncogenes by aberrant SHM (e.g., *PIM1, PAX5, RHOH, MYC, BTG2, KLHL14, SUSD2*), translocations of the IG and *BCL6* genes, and genomic instability (e.g., gains of 18q21; losses of 9p21, 8q12, 6q21) occur in these cells in the course of their malignant transformation. Activated Toll-like receptor, B cell receptor (BCR), and NF-κB signaling pathways foster lymphoma cell proliferation. Hence, tumor cells are arrested in a late B cell differentiation stage, corresponding to late GC exit B cells, which are genetically related to IgM+ memory cells. Paradoxically, the GC reaction increases self-/polyreactivity, yielding increased tumor BCR reactivity for multiple CNS proteins, which likely contributes to CNS tropism of the lymphoma. The loss of MHC class I antigen expression supports tumor cell immune escape. Thus, specific and unique interactions of the tumor cells with resident CNS cells determine the hallmarks of PCNSL.

## 1. Historic Background

For a long time, the exclusive manifestation of malignant lymphoma in the central nervous system (CNS) has remained mysterious and has been frequently debated. Indeed, in the early 20th century, the tumor classified today as primary CNS lymphoma (PCNSL) [1,2] or, in the upcoming revised WHO Classification of Tumours of the Nervous System, as primary CNS diffuse large B cell lymphoma (DLBCL, PCNS-DLBCL), was suggested to be related to sarcoma [3]. This view relied on the morphological observation of tumor cells intimately intermingled with argyrophilic fibers, which are part of cerebral blood vessel walls. The identification of the hematogenous nature of this lymphoma was not achieved before the development of the Kiel classification of lymphomas, Ref. [4] which, based on morphological similarities, identified tumor cells as centroblasts or, less frequently, as centrocytes and immunoblasts. Advances in immunological and molecular genetic techniques have allowed the precise characterization of tumor cell pheno- and genotypes as well as their classification, and now provide the rationale for the current diagnostic strategy defined according to the WHO classification of CNS and hematopoietic tumors [1,2]. The term “PCNSL” has been modified to “PCNS-DLBCL” to precisely define this entity; this terminology appreciates the specific nature of the target organ, i.e., the immunoprivileged CNS, and also delineates PCNS-DLBCL from other lymphoma entities that may be present in the CNS. The latter mainly include low-grade B cell lymphomas, mucosa-associated lymphatic tissue (MALT) lymphoma of the dura, and various immunodeficiency-related hematopoietic lesions, all of which may manifest in the CNS either exclusively or as part of a generalized disease. Clinically, PCNS-DLBCL needs to be distinguished from the CNS manifestation of extracerebral DLBCL. We propose that the term “PCNSL” should be reserved for the most common (and probably only) molecular subtype of PCNS-DLBCL, namely, PCNS-DLBCL with MYD88 and B cell receptor (BCR) signaling pathway mutations, which is nowadays described as the MCD/C5/MYD88 molecular group (see below). For practical reasons, current diagnostic work-up includes (1) the phenotypic identification of the tumor cells of PCNSL as late germinal center (GC) exit B cells and (2) careful clinical staging to exclude the possibility of extracerebral DLBCL. In unequivocal cases, this algorithm correctly identifies PCNSL corresponding to the MCD/C5/MYD88 DLBCL subgroup when investigated by sequencing technologies.

## 2. Molecular Pathogenesis of PCNSL

Historically, deciphering the pathogenesis of PCNSL has been severely hampered by the small size of stereotactic biopsy samples, which generally restrict experiments to addressing one single question. This natural limitation has necessitated the stepwise systematic analysis of biopsies by either immunohistochemistry, fluorescence in situ hybridization, or single-gene PCR and RT-PCR with subsequent sequencing for decades. A major step forward was the introduction of arrays that allowed genome-, epigenome-, and transcriptome-wide studies. Thus, intense research in the last two decades has succeeded in shedding light on the molecular pathogenesis of PCNSL in immunocompetent patients. Since the pathogenesis of PCNSL in immunodeficient patients is fundamentally different (with the vast majority of cases being related to the Epstein–Barr virus (EBV) infection [5]), we have focused on immunocompetent patients in whom PCNSL is, with rare exceptions, EBV-negative [5,6]. Using this strategy, we and others have provided answers to the most important open questions arising from the peculiarities of the malignant lymphoma entity of PCNSL:

(1) What is the cellular origin of these tumor cells?
(2) What are the pathogenetically relevant genetic, epigenetic, transcriptional, and proteomic alterations of these tumor cells?
(3) What are the reasons for the tropism and exclusive manifestation of this tumor in the CNS?

The integration of various experimental data has provided strong evidence for the hypothesis that the clue to PCNSL lymphomagenesis lies in the developmental processes of the cell of origin. Here, we summarize these processes and their impact on the target organ, i.e., on the CNS, on tumorigenesis, and on the tumor cell molecular genotype and phenotype, which directly translate into neuropathological diagnostic features and criteria. In the long run, these data may serve as a basis for the creation of individualized, targeted therapy, hopefully improving patients’ prognosis and quality of life.

**Figure 1:**
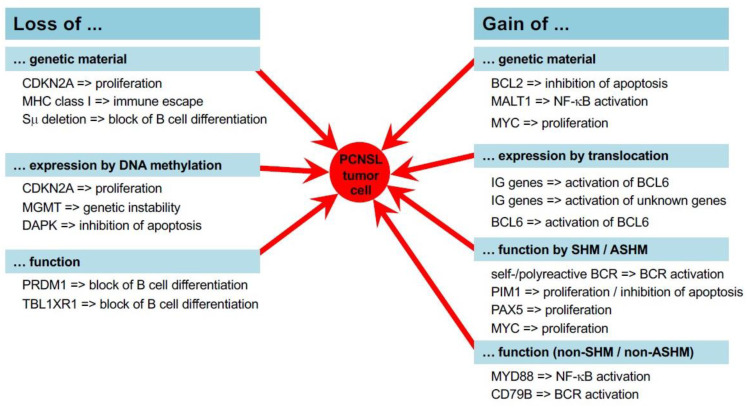
Molecular mechanisms of PCNSL pathogenesis. In PCNSL, several mechanisms such as DNA repair, distinct methylation programs, the process of somatic hypermutation, and distinct expression programs are altered and/or error-prone. Ultimately, this leads to gains and losses of genetic material and the aberrant expression of genes activating proliferation and survival fostering pathways, i.e., the TLR, BCR, and NF-κB pathways.

Since recurrent molecular alterations have been reviewed in detail elsewhere [7,8,9,10], here we restrict our discussion of this aspect to a brief summary. The hallmarks of PCNSL are genetic, epigenetic, and transcriptional alterations (summarized in Table 1) that converge to support and sustain survival by fostering proliferation and impairing prognosis (Figure 1).

**Figure 2:**
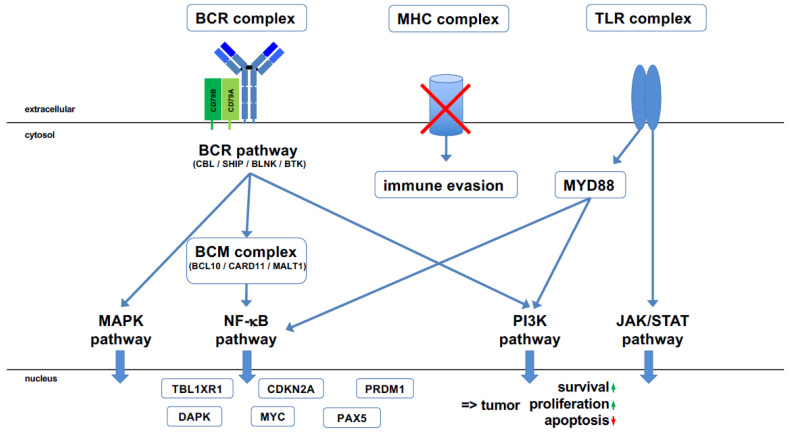
Major pathways involved in PCNSL pathogenesis. Various molecular alterations converge on the B cell receptor (BCR) pathway, which is tonically activated. This is complemented by the Toll-like receptor (TLR) pathway, which is activated by mutated *MYD88*, a master signaling instance of the TLR pathway. *CD79B*, encoding part of the BCR complex, is the second most frequently mutated gene in PCNSL. Several other genes that regulate the BCR pathway are mutated in a characteristic manner; genes with an inhibitory effect are rendered non-functional and genes activating the BCR pathway are constitutively activated. The BCM complex, consisting of BCL10, CARD11, and MALT1, functions as a gatekeeper of the NF-kB pathway. *CARD11* is recurrently mutated; *MALT1* is part of the recurrent 18q21 amplification, often together with the anti-apoptotic BCL2 gene. A hallmark of PCNSL is an activated NF-κB pathway, although the genes of the NF-κB pathway are not recurrently mutated. Tonic BCR signaling activates at least three different pathways, including the NF-κB pathway, the PI3 kinase (PI3K) pathway, and the MAP kinase (MAPK) pathway. TLR signaling also activates the NF-κB pathway, the PI3K pathway, and the JAK/STAT pathway. The tumor suppressor gene *CDKN2A*, which encodes p14ARF and p16INK4a, is rendered non-functional in PCNSL through either mutation or methylation. *TBL1XR1* and *PRDM1* mutations are responsible for a shift in B cell differentiation to an IgM memory B cell-like phenotype. The synergistic effect of the various activated pathways, together with mutations in distinct genes and the constitutive expression of important transcription factors for B cell differentiation, including MYC and PAX5, results in tumor cell survival with increased proliferation and blocked apoptosis. In addition, a loss of MHC class I facilitates immune evasion in an immunoprivileged organ.

**Figure 3:**
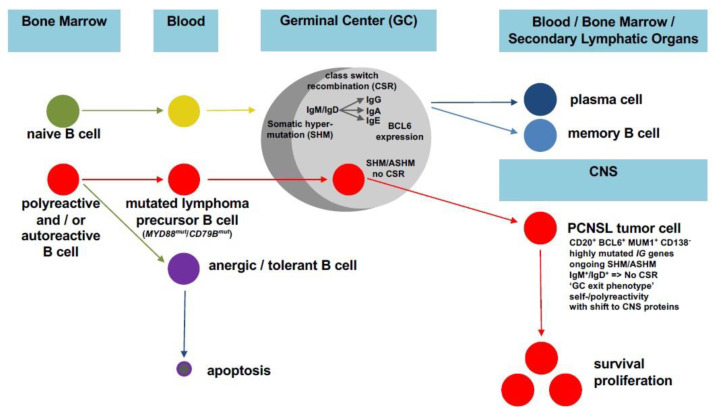
Model of the impact of a faulty GC reaction on PCNSL pathogenesis. Instead of normal B cell maturation with the SHM of a naïve B cell resulting in a terminally differentiated plasma/memory B cell (upper panel), (a) mutation(s) prevent(s) the apoptosis of a self-/polyreactive B cell, thus conferring a survival advantage. Upon GC entry, SHM starts, leading to the modification of the IG and *BCL6* genes. Through the aberrant extension of SHM to proto-oncogenes (aberrant SHM, ASHM) and the occurrence of translocations, the B cell acquires further oncogenic hits while simultaneously becoming unable to terminate SHM. This vicious cycle increases self-/polyreactivity without being (further) selected for high-affinity BCR antigen binding, thus resulting in a PCNSL (precursor) B cell characterized by a late GC exit phenotype without an IG class switch, thus being related to IgM+ memory B cells with self-/polyreactivity and a shift towards the recognition of increased numbers of proteins that are physiologically expressed in the CNS.

The integration of these molecular data obtained from various individual studies converges on the activation of the Toll-like receptor (TLR)-, BCR-, and NF-κB signaling pathways in PCNSL (Figure 1 and Figure 2). Genetically, PCNSLs segregate along the entire spectrum of the ABC and GCB sub-types defined for systemic DLBCL and exhibit further features associated with ABC-DLBCL [6,20,23,30,31,32]. Thus, these studies identify the tumor cells of PCNSL as antigen-experienced with a prolonged stay in the GC and the characteristics of IgM+ memory B cells (Figure 3) [6,30].

Until the year 2012, the key hallmarks of the genomic and transcriptional landscape of PCNSL—including frequent *MYD88* and BCR mutations, chromosomal translocations, MHC deletions, aberrant SHM, patterns of genomic imbalances, and a GC exit profile—have been fully established by work carried out by us and others using hypothesis-driven targeted analyses (Figure 1 and Figure 2) [6,13,15,16,20,23,24,26,27,28,30,31,32,33,34,35,36,37]. The technical advances introduced thereafter have allowed us to carry out comprehensive in-depth analyses at both the RNA and DNA level with a high sensitivity, thus circumventing the problem of the limited amount of tissue available for analysis [22,38,39,40,41]. Notably, recent studies that have taken advantage of this technical progress have fully confirmed all prior findings [22,29]. Aiming at the identification of targetable genetic features, PCNSLs were “re-invented”, yielding an 18q gain; *PRDM1*, *PIM1*, *BTG1*, and *TBL1XR1* mutations; aberrant SHM; an ABC-DLBCL genotype; and MHC class I deletions. These findings perfectly confirmed previous observations and the hypothesis that the tumor cells of PCNSLs are characterized by unique genetic, transcriptional, and phenotypic features.

Interestingly, four independent studies [42,43,44,45] that analyzed large series of de novo DLBCL irrespective of their site of manifestation identified a genetic subgroup termed MCD, C5, or MYD88 that perfectly matched the characteristics of PCNSL, with *MYD88* and *CD79B* mutations; BCR-dependent NF-κB activation; an ABC profile; *CDKN2A*, *ETV6*, *BTG1/2*, and *TBL1XR1* genetic alterations; and a high expression of *MYC*-induced genes. Importantly, lymphomas with these features turned out to be derived from the CNS and testis, which are both immunoprivileged organs [42,44]. One of these studies that used whole-genome rather than exome sequencing also corroborated the existence of a mutationally defined MYD88 group among DLBCLs; interestingly, this study also pointed to some variability in cluster assignment and the existence of additional genetic subgroups if the mutation classification was extended to other GC-derived lymphomas [45]. Of great clinical importance, the MCD/C5/MYD88 molecular subtype identified in systemic DLBCL has been associated with the worst clinical outcomes out of all the molecular subtypes [43], and other studies have reinforced the longstanding clinical observation of a worse prognosis of PCNSL as compared to extracerebral DLBCL [1,2]. These studies have raised hopes that patients might benefit from the therapeutic targeting of the BCR signaling pathway and/or immune checkpoint inhibition. To this end, BCR signaling has been targeted by ibrutinib, a BTK inhibitor, in a limited number of patients [46,47]. The addition of ibrutinib to high-dose methotrexate (HD-MTX) and rituximab in 15 patients with recurrent/refractory PCNSL yielded a clinical response in 12 of them (80%) [46]. In another study, the addition of ibrutinib to an HD-MTX regimen in 52 patients with recurrent/refractory PCNSL achieved a clinical response in 32 patients (62%) [47]. The idea that a therapeutic approach addressing immune checkpoint inhibition in PCNSL may be attractive is based on the observation that gains of 9p24.3 (*PDL-1*/*PDL-2*) have been identified in PCNSL. However, the frequency of these gains varies markedly in independent studies, ranging from 21% (4/19) [27] to 67% (28/42) [22]. Furthermore, a variable expression of PDL-1/PD-1 protein has been reported, and it remains unknown whether molecular aberrations correlate with the protein level (reviewed by Wirsching et al. [48]). This raises questions regarding the technological approaches and cut-offs used to diagnose these aberrations. In addition, these data should encourage studies to be carried out in larger numbers of patients to reveal their real frequency more precisely, and further studies are needed to clarify the potential clinical relevance of immune checkpoint inhibitors in the treatment of PCNSL.

Overall, while the reported alterations converge on final common signaling pathways, it is of note that distinct molecular changes yield functionally similar results. Thus, as PCNSL are not genetically identical, the addition of therapeutic regimens targeting specific genes requires the genetic analysis of tumor biopsies as a rationale for personalized PCNSL therapy.

## 3. A Faulty GC Reaction as Key Process for PCNSL Pathogenesis

The key to PCNSL lymphomagenesis is the GC reaction [49]. Physiologically, B cell development corresponds to precisely defined molecular modifications of the IG genes in defined organs of the immune system, yielding mature B cells that characteristically recognize a specific, unique antigen with a high affinity via their BCR [50]. Briefly, the bone marrow progenitors of B cells first rearrange the V, D, and J segments of the IG heavy (IgH, 14q32.33) chain loci and then the V and J genes of their light chain loci (Igλ, 22q11.2, Igκ 2p11.2) are rearranged as pre B cells. After light chain rearrangement and the first expression of a BCR as an IgM molecule at the immature B cell stage, counterselection takes place, leading to strong self-reactivity. B cells with autoreactivity are either edited (new light chain rearrangements) or eliminated by apoptosis. After successfully expressing a BCR without (strong) self-/polyreactivity, the respective B cells leave the bone marrow to circulate through the peripheral blood and secondary lymphatic organs as naïve, mature B cells. Antigen encounter together with T cells help initiate the GC reaction, which aims at increasing the affinity of the BCR for its specific antigen. The molecular process that builds the basis for affinity maturation is the process of SHM, through which point mutations and some indels are introduced into rearranged IG heavy- and light-chain V genes, starting downstream of the promoter of rearranged IGV genes. In addition to this process of SHM, many cells also undergo IG CSR in the GC. CSR corresponds to the replacement of the µ and δ IG heavy-chain constant region genes of the BCR with one of the constant-region genes located downstream to generate IgG, IgA, or IgE antibodies with distinct effector functions. As the complex processes of SHM and CSR require the introduction of DNA strand breaks at the respective loci combined with error-prone repair mechanisms, GC B cells are highly vulnerable to oncogenic events [51]. Only B cells that have successfully passed these complex processes, usually after multiple rounds of proliferation, mutation, and selection are allowed to terminally differentiate into memory B cells or plasma cells; if not, they undergo apoptosis (Figure 3). Thus, there are several major checkpoints of B cell differentiation before the entrance, inside and at the exit of the GC.

The failure of correct selection processes during the GC reaction can result in lymphoma development [52]. This is likely also the scenario causing PCNSL. PCNSL cells frequently use the *IGHV4-34* gene (55–80% of cases), which often encodes autoreactive antibodies [6,11,12]. Although, physiologically, B cells expressing the *IGHV4-34* gene lose autoreactivity if involved in a GC reaction as a result of SHM, this does not occur in the precursor cells of PCNSL. Instead, they continue to exhibit self- and/or polyreactivity [53,54,55,56], as shown by experiments in which the BCR of the tumor cells was reverted into the BCR of the corresponding naïve B cell [56]. With the progression of the BCR from naive to mutated, the BCR of PCNSL increases its self-/polyreactivity [56]. Interestingly, among the antigens recognized by such BCR, a high number of proteins are physiologically expressed in the CNS [56]. The escape of the self-/polyreactive precursor cell from cell death may be supported by mutations occurring early in their development [29] that facilitate B cell survival. In this regard, the high frequency of *MYD88* (36–85% of PCNSL cases) [20,21,41] and *CD79B* (8–59% of PCNSL cases) [21,23,41] mutations is of note because they foster signaling along the TLR and BCR pathways, respectively (Figure 1 and Figure 2). In line with the idea that MYD88 and CD79B mutations play a major role in the early stages of PCNSL pathogenesis, these mutations are typically clonal events with a high variant allele frequency, indicating their early occurrence.

The combination of a putative autoreactive BCR with already-acquired apoptosis-suppressing genetic lesions may also be the main reason for the unusually high mutation load in the IGHV gene of PCNSL; these factors promote an elongated GC reaction, resulting in multiple rounds of proliferation and mutation. In addition to SHM, tumor cells or their precursors have also been shown to initiate the process of CSR [15], which have however failed due to internal sµ deletions resulting in tumor cells with a fixed IgM/IgD geno- and phenotype [15]. The reason for the incomplete CSR is not clear, but it has been speculated that this may be of advantage for GC-associated lymphoma cells, as human IgM+ memory B cells have a propensity for repeated GC reactions upon re-activation, whereas IgG+ memory B cells preferentially differentiate into (resting) plasma cells upon re-stimulation [57,58]. Thus, “freezing” B cells at the IgM isotype may be more compatible with prolonged and/or repeated GC reactions, which promote malignant transformation.

DNA strand breaks that are mechanistically involved in internal sμ deletions during CSR attempts as well as in SHM are likely to be the major cause of translocations involving the IG loci, which are seen in about 30–40% of PCNSLs [7,19]. Notably, PCNSLs also frequently harbor translocations involving the *BCL6* gene (13–47% of cases) [7,19,22,59]. It is likely that these are also mediated through the activity of the SHM process, as *BCL6* is a main off-target of SHM and the region targeted by SHM is the same one as where *BCL6* translocation breakpoints are found. Remarkably, *MYC*, *BCL2*, and *MALT1*, which are frequently involved in translocations in extracerebral DLBCL [60], are not targeted in PCNSL [7,16]. While many translocation partners of the IG genes—except for *BCL6* [16,17] and, recently, *PD-L2* and *JAK1* as partners of the Igλ and IgH loci, respectively [22]—could not yet be identified, the promiscuous *BCL6* gene fuses with *IGH*, *IGL*, *HSP90A*, *GAPDH*, *H4C9*, and *LPP* [16,17,18]. The simultaneous expression of MYC and BCL6 is required for cyclic re-entry into the GC [61]. The expression of MYC together with ongoing BCL6 activity and deleterious *PRDM1* [24,29] and *TBL1XR1* mutations [22,25,29] may foster the cyclic reentry of the GC, thus further supporting the notion of the aberrant SHM of genes with oncogenic potential, including *PIM1*, *MYC*, *RHOH*, *PAX5*, *KLHL14*, and *SUSD2*, as well as members of the large histone cluster on 6p21 [13,14]. Furthermore, *TBL1XR1* mutations originally detected by Gonzalez-Aguilar et al. [25] in three of 21 PCNSL (14%) may also contribute to survival, since they can lead to pre-memory transcriptional reprogramming and cell-fate bias [62,63]. Collectively, a vicious cycle results in an IgM+ B cell that is genetically related to memory B cells; however, it will be trapped in a GC reaction and therefore unable to complete the process of differentiation into resting post-GC memory B cells or plasma cells (Figure 3).

## 4. Tumor Cell Adaptation to the CNS Microenvironment in PCNSL

As detailed above, PCNSL tumor cells or their precursors participate in a prolonged, even ongoing GC reaction with active SHM. In addition to its oncogenic impact, this process further impacts on tumor cells since they fail to increase their affinity for a unique antigen [53]. Instead, these cells increase their self-/polyreactivity, yielding low-affinity B cells [56]. Compared to naïve B cells, tumor cell BCRs recognize significantly higher numbers of proteins, as shown in protein microarray and immunoprecipitation studies [56]. Increased polyreactivity goes hand in hand with an increased reactivity of the tumor cell BCR with proteins expressed in the CNS. Instead of narrowing down the BCR reactivity for a single antigen recognized with a high affinity, tumor cells broaden their antigenic repertoire for CNS proteins and gain the ability to interact with a plethora of CNS proteins, which fosters their proliferation and survival [56]. This perverted B cell differentiation yields a tumor cell that is perfectly adapted to the CNS microenvironment. Thus, in this scenario, B cell differentiation links specific oncogenesis-induced features of tumor cells with the microenvironment of the CNS. This misled differentiation explains, at least in part, the particular tropism of malignant B cells for the CNS as their unique target organ.

**Figure 4:**
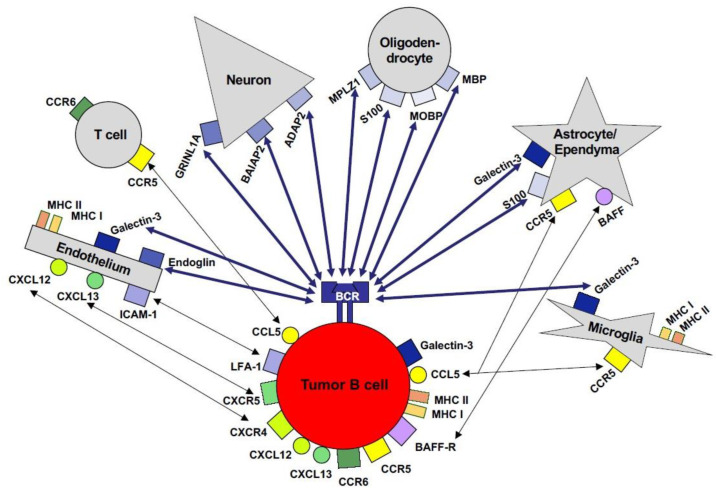
Cellular interactions of PCNSL tumor cells in the microenvironment of the CNS. The malignant B cells of PCNSLs may interact with cells in the CNS in both a BCR-dependent as well as a BCR-independent manner. A plethora of proteins physiologically expressed by neurons, astrocytes, ependyma, microglia/macrophages, and/or endothelial cells can be recognized by the BCR of self-/polyreactive B cells. BCR-independent interactions with resident CNS cells and reactive inflammatory T cells may be mediated via chemokines, cell adhesion molecules, and their respective receptors.

Regarding potential BCR-mediated interactions in the CNS, tumor and/or precursor cells may interact with CNS proteins in a cell-type-specific manner with various resident CNS cell populations (Figure 4) [53,54,55,56]. Physiologically, neurons express GRINL1A, BAIAP2, ADAP2 (centaurin-α) [53], and SAMD14 [55]; MPLZ1, MBP, and MOBP are constituents of myelin/oligodendrocytes [53], and S100 protein family members are expressed by astrocytes and oligodendrocytes [53]. Under pathological conditions, including tumor infiltration and inflammation, CNS cells upregulate further proteins recognized by the BCR of the tumor cells. For example, cerebral endothelial cells express endoglin and galectin-3; galectin is also induced in activated astrocytes and microglia/macrophages (Figure 4) [53]. The expression pattern of proteins that may serve as antigens for the binding of tumor cells via their BCR may also contribute to the preferential growth of PCNSL in the deep grey and white matter of the cerebral hemispheres.

Thus, with these lymphoma cell characteristics and CNS proteins as potential antigens for their BCR as well as the presence of CD4 T cells and antigen-presenting cells in the brain, the basic requirements for a GC reaction are fulfilled in the CNS, which lacks a conventional lymphatic drainage system. In addition to physiologically expressed proteins, infectious pathogens are also candidates for triggering the BCR of PCNSLs. In this regard, viruses with a CNS tropism and the capacity to persist in the brain are of interest, including viruses of the herpes family and polyoma-viruses. Thus far, no specific pathogen that may play a role in PCNSL lymphomagenesis has been identified. Interestingly, the restricted pattern of IGHV gene segments together with the random usage of IGHD and IGHJ genes would be compatible with a superantigen effect. However, the candidates for superantigens involved in PCNSL pathogenesis are still unknown. Further-more, the intriguing question of whether a GC reaction indeed occurs in the CNS has not yet been resolved. In some patients with chronic multiple sclerosis, clusters of activated B cells have been identified in the leptomeninges overlying multiple sclerosis lesions and these have been termed “tertiary lymphoid follicles” [64]. However, definitive evidence that SHM indeed occurs in these clusters is still lacking. In this regard, it is of note that such structures resembling follicular structures are absent from PCNSL in both parenchymal tumors as well as adjacent leptomeninges. Thus, the important question of whether malignant transformation occurs within or outside the CNS still remains to be answered.

In addition to BCR-mediated interactions between tumor cells and resident brain cells, cell–cell interactions between tumor cells and CNS cell populations may also support lymphoma dissemination, with the widespread and multifocal involvement of the brain parenchyma thus being prognostically relevant. For instance, tumor cells can recognize galectin-3 via their BCR as well as independently of the BCR (Figure 4). Interestingly, in 25% (5/20) of PCNSLs, the tumor cells themselves express galectin-3, which binds to CNS glycans expressed by microglia/macrophages including MAC1, MAC3, and CD45 [53]. Regarding cell adhesion molecules and chemokines, no pattern selectively expressed by the tumor cells has yet been reported [65]. Nevertheless, cell–cell interactions via cell adhesion molecules and chemokines and their receptors may play a role in intracerebral lymphoma dissemination. For example, LFA-1+CXCR4+CXCR5+ tumor cells may interact with ICAM-1+, CXCL12+/CXCL13+ endothelial cells, and ICAM-1+CXCL13+ microglia (Figure 4) [66]. Thus, a unique pattern of interactions via various cell-surface molecules results from their reciprocal, differential, and cell-type-specific expression in resident CNS cell populations (Figure 4). In addition to direct cell–cell interactions, tumor cells and the cells of their microenvironment may also communicate via a plethora of soluble mediators, with the network involving the differential contribution of various players still remaining to be described in detail.

Moreover, escape from an immune reaction in the already “immunoprivileged” CNS further contributes to target organ tropism, widespread dissemination, and adverse prognosis in PCNSL [27,33]. As early as the year 2000, Kluin and co-workers reported a loss of MHC class I and II genes, including β2-microglobulin as hallmarks of DLBCL of the CNS and testis, both of which are immunoprivileged organs [33]. The recurrent loss of the MHC class I gene due to 6p21 deletions (7/19, 37%) allows tumor cells to escape a potential CD8 T cell immune response [26,27,33]. Furthermore, monoallelic MHC alterations or partial uniparental disomies, as additional mechanisms of immune escape commonly found in DLBCLs [67], were also detected in 37% (7/19) of PCNS-DLBCLs studied [27]. In fact, in the CNS, the tumor cells are admixed with reactive CD4 and CD8 T cells and B lymphocytes, the functional role of which still remains to be defined [68,69,70,71]. Interestingly, the analysis of the cerebrospinal fluid proteome signature of PCNSL patients identified an enrichment of the extracellular part of HLA-DRB1, which may be due to the matrix metalloproteinase-mediated ectodomain shedding of MHC class II molecules from tumor cells [72]. This partial loss of MHC class II antigens may further contribute to the immune escape of tumor cells. With these unique features, i.e., interaction-induced tumor cell proliferation together with immune escape in the immunoprivileged CNS, tumor cells gain an important survival advantage in the CNS with reciprocal cell–cell interactions at multiple levels.

Regarding growth, the strong MYC expression of malignant B cells may provide an additional benefit for dissemination in the CNS. Similar to PCNSL, Burkitt lymphoma, which is defined by a high MYC expression, has a high affinity for the CNS [73]. In a preclinical murine model of PCNSL and Burkitt lymphoma, Myc+ tumor cells spread throughout the brain parenchyma in a pattern resembling human PCNSL (unpublished own data).

Thus, there is mounting evidence that self-/polyreactive B cells à priori destined to undergo apoptosis receive MYD88 and CD79B mutation-induced TLR- and BCR-mediated stimuli supporting their survival as well as anti-apoptotic stimuli; thus, advantageously, B cells become able to bypass elimination prior to their entrance into the GC. Instead, the cells enter the GC, where a faulty or ongoing GC reaction increases their self-/polyreactivity for CNS antigens (Figure 3). This contributes to target organ tropism and the spread of the cells throughout the brain, as well as providing oncogenic stimuli (translocations, gains/losses of genetic material, aberrant SHM of oncogenes). Finally, the highly proliferatively active tumor cells become trapped in a vicious GC cycle and are unable to terminally differentiate.

## 5. Role of Preclinical Animal Models in Studying PCNSL Pathogenesis

**Figure 5:**
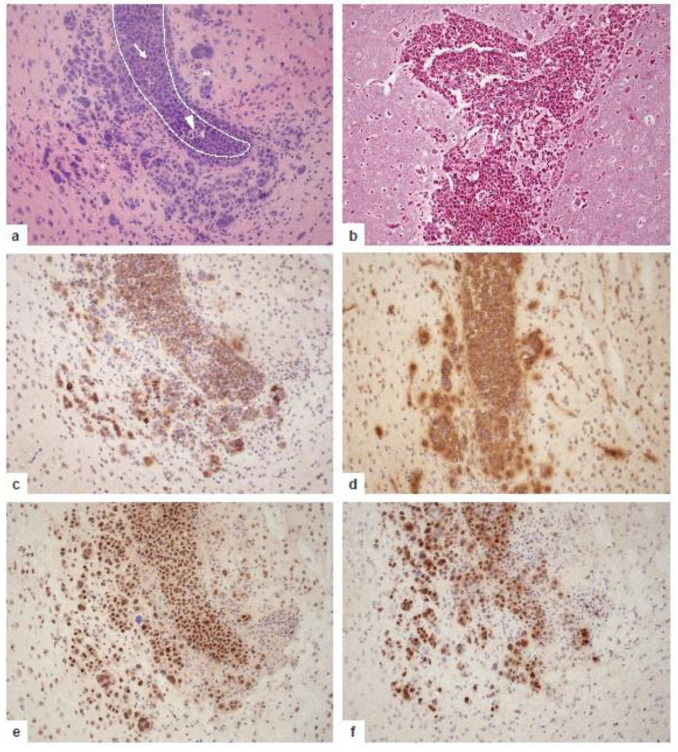
Growth pattern and phenotypic characteristics of murine PCNSL at day 16 after the transplantation of 5 × 10^5^ BAL17^CNS^ cells into the right frontal lobe of BALB/C mice. (**a**) Highly cellular lymphoma in the lumen of the prominently enlarged lateral ventricle with small foci of necrosis (arrowhead); arrow points to a mitotic figure. The destruction of the ependymal border of the lateral ventricle with groups of tumor cells infiltrating the adjacent cingulate cortex, the corpus callosum, and the striatum. The tumor cells are associated with many lymphocytes and activated microglia (**). Dotted line indicates the border of the lateral ventricle invaded and destroyed by the tumor cells. Hematoxylin and eosin staining. (**b**) Angiocentric growth pattern of PCNSL with splitting of blood vessel walls by the tumor cells. Reticulin silver impregnation. (**c**) CNS lymphoma cells express B cell markers including CD19 and B220. Immunohistochemistry with rat anti-CD19 (clone 6D5, Abcam, Cambridge, UK) and rat anti-B220 antibodies (clone RA3-6B2, Abcam). (**d**) BAL17^CNS^ lymphoma cells show the cell surface expression of IgM. Immunoreactivity of some capillaries and their vicinity indicates edema. Immunohistochemistry with polyclonal goat anti-IgM (Southern Biotech, Birmingham, AL, USA). (**e**) BAL17^CNS^ lymphoma cells strongly express the c-MYC protein. Immunohistochemistry with rabbit anti-c-MYC antibody (clone Y69, Abcam). (**f**) The high proliferative and mitotic activity of BAL17^CNS^ lymphoma cells is evidenced by the strong nuclear expression of the Ki-67 antigen in >90% of the tumor cells. Some activated microglial cells and small to slightly enlarged reactive lymphocytes are also Ki-67+, indicating their strong activation. Immunohistochemistry with rabbit anti-MIB-1 antibody (clone SP6, DCS Diagnostics, Hamburg, Germany). (**a**–**d**) original magnification ×200.

**Figure 6:**
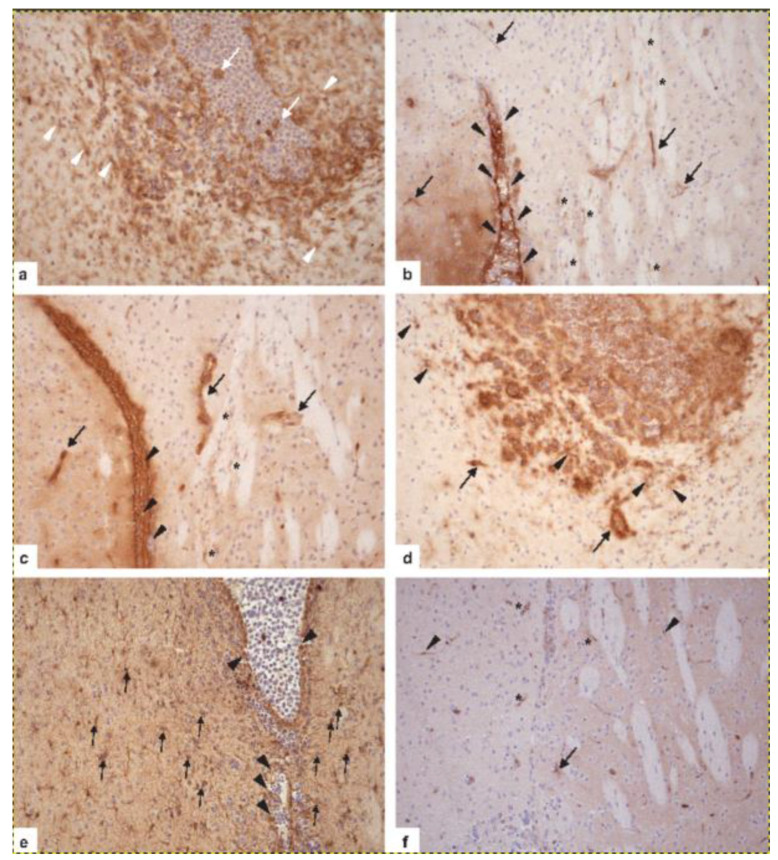
Activation of resident CNS cell populations in murine PCNSL at day 16 after the transplantation of 5 × 10^5^ BAL17^CNS^ cells into the right frontal lobe of BALB/C mice. (**a**) In the vicinity of BAL17^CNS^ lymphoma, CD11b/c+ microglial cells in the cingulate cortex, the corpus callosum, and the striatum are prominently activated as evidenced by long, elongated, ramified processes (arrowheads). Note that activation as indicated by morphology, i.e., staining intensity with the anti-CD11b/c antibody and ramification of processes, increases with the proximity to the lymphoma. Some CD11b/c+ macrophages are present within bulk lymphoma (arrows). Immunohistochemistry with anti-Mac-1α (C3bi receptor) antibody (ATCC, clone M1/70.15). (**b**) ICAM-1 is expressed on ependymal cells of the lateral ventricle (arrowheads) and on BAL17^CNS^ lymphoma cells in the lumen of the lateral ventricle (white *). Cerebral endothelial cells of small capillaries in the cingulate cortex and the caudate putamen have upregulated ICAM-1 (arrows). Here, some microglial cells also have induced ICAM-1 (black *). Immunohistochemistry with hamster anti-CD54 (ICAM-1) antibody (clone 3E2, BD Biosciences, Heidelberg, Germany). (**c**) MHC class I antigen is upregulated on cerebral endothelial cells (arrows) and microglia (*) close to the lateral ventricle, which harbors BAL17^CNS^ lymphoma cells (arrowheads). Immunohistochemistry with anti-H-2 antibody (all haplotypes, ATCC, clone M1/42.3.9.8 HLK). (**d**) MHC class II antigen is expressed on a fraction of BAL17^CNS^ lymphoma cells in the lumen of the lateral ventricle and the adjacent cingulate cortex and corpus callosum, on cerebral endothelial cells (arrows), and on strongly activated microglia (arrowheads). Immunohistochemistry with anti-I-A antibody (b,d,q haplo-types, ATCC clone M5/114.15.2). (**e**) Lymphoma cells have focally destroyed the ependymal wall of the lateral ventricle (arrowheads) and have invaded the adjacent periventricular tissue. The prominent activation of GFAP+ astrocytes (arrows) intermingled with tumor cells. Immunohistochemistry with polyclonal rabbit anti-GFAP (Agilent, Santa Clara, CA, USA). (**f**): In the vicinity of lymphoma cells, cerebral endothelial cells (*), an astrocyte (arrow), and microglial cells (arrowheads) have upregulated galectin-3. Immuno-histochemistry with rabbit-anti-galectin-3 (clone EP27754, Abcam). (**a**–**f**) Original magnification, ×200.

The major contribution of animal models to deciphering the pathogenesis of PCNSL is to precisely dissect the interactions occurring between tumor cells and individual components of the CNS microenvironment, which can provide important insights into fundamental neuroimmunological mechanisms far beyond PCNSL lymphomagenesis. This long-term goal requires the system of an immunocompetent, syngeneic host. Two attractive murine models that fulfill these requirements have been developed. Donnou et al. [74] used A20.IIA-GFP cells in a model of immunocompetent BALB/c mice in which lethal CNS lymphoma had been induced. In addition to IgG+ A20.IIA-GFP cells that have successfully performed CSR and are thus more mature than the tumor cells of PCNSL, the model of BAL17^CNS^-induced PCNSL using IgM+ lymphoma cells adapted to the CNS microenvironment resembles PCNSL more closely [75]. Vigorously proliferating B220+CD19+IgM+ BAL17^CNS^ cells expressing Myc derived from BAL17 lymphoma cells by repeated isolation and re-transplantation into the CNS [75] disseminate in the CNS in a topographical manner similar to that of human PCNSL with preferential growth in the ventricular system, the adjacent basal ganglia, and hemispheric white and grey matter; they also exhibit the characteristic angiotropism (Figure 5a–f) [75]. In both the A20.IIA-GFP and BAL17^CNS^ lymphoma models, CD4+ and CD8+ T cells, reactive B cells, and increased numbers of CD11b+ and CD11c+ cells intermingle with tumor cells (Figure 6a). Interestingly, in BAL17^CNS^ PCNSL, the activation of resident brain cells was locally confined to areas of tumor infiltration. Microglia and cerebral endothelial cells reacted with an upregulation of ICAM-1 and MHC class I and II antigens (Figure 6b–d), thus meeting the requirements for an anti-tumor T cell immune response. Astrocytes upregulated GFAP and the S100 protein [75] and, similar to endothelial cells and microglia, galectin-3 (Figure 6e,f). These data further illustrate how, in principle, the malignant lymphoma cells of PCNSLs may interact with resident brain cells. The further precise dissection of this finely tuned neuroimmune network using genetically modified mice will have an enormous impact on our understanding of the role of the unique CNS microenvironment on PCNSL pathogenesis. Furthermore, the functional role of the individual molecular alterations identified in human PCNSL biopsies and, potentially, their therapeutic impact can be dissected precisely in these preclinical models, thus ideally complementing tumor tissue analyses.

## 6. Neuropathological Diagnostics of PCNSL

**Figure 7:**
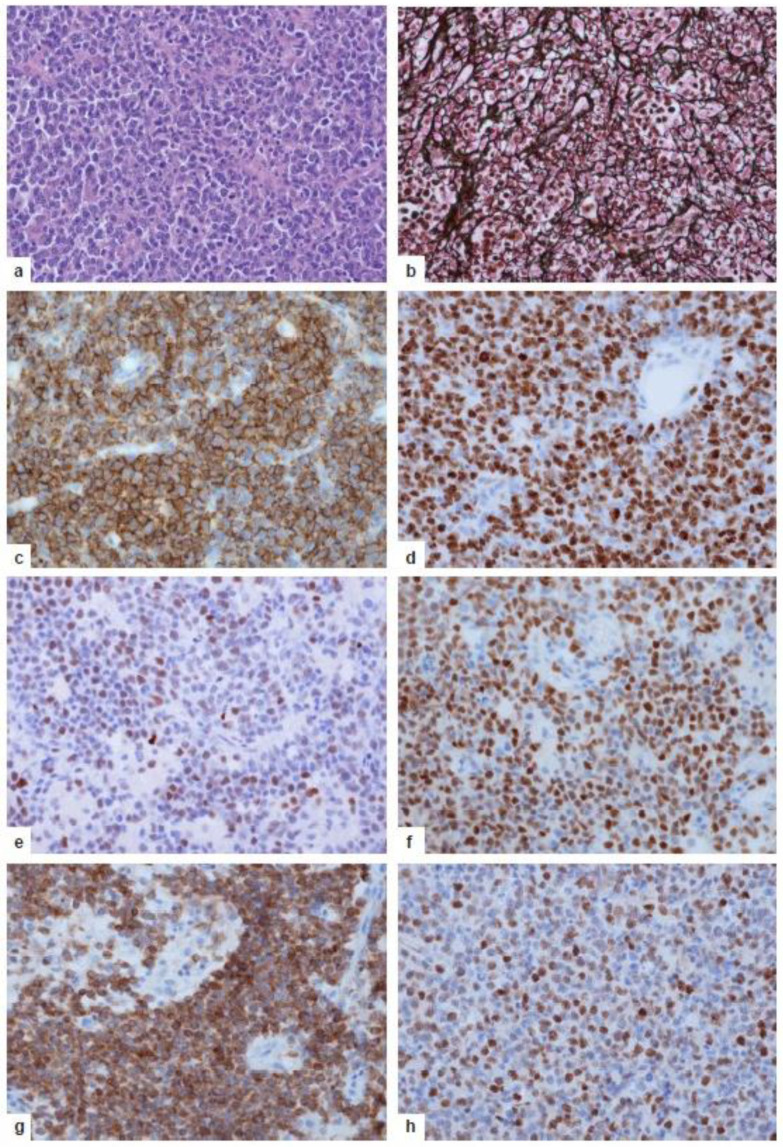
Neuropathology of human PCNSL. (**a**) Highly cellular tumor with patternless growth. Hematoxylin and eosin staining. (**b**) Angiocentric infiltration pattern of the tumor cells. Fragmentation of the argyrophilic fibers of blood vessel walls by the tumor cells, which are surrounded by reticulin fibers. Reticulin silver impregnation. (**c**) The tumor cells express the pan-B-cell marker CD20 (mouse anti-CD20, clone L26, DCS Diagnostics). (**d**) High proliferative activity of the tumor cells, with >90% expressing the Ki-67 antigen (rabbit anti-MIB-1, clone SP6, DCS Diagnostics). (**e**,**f**) The majority of the tumor cells exhibit a late GC exit phenotype with the expression of the BCL6 (d, rabbit anti-BCL6, clone EPR11410-43, Abcam) and MUM1/IRF4 (e, clone rabbit anti-MUM1, clone BC5, Zytomed Systems, Eching, Germany) protein. (**g**,**h**) The majority of tumor cells are characterized by a combined strong expression of BCL-2 (g, mouse anti-BCL2, clone bcl-2/100/D5, DCS Diagnostics) and c-MYC (>41%, h, rabbit anti-c-MYC, clone Y69, Abcam). (**b**–**h**) Immunohistochemistry with the respective primary antibody detected by an appropriate secondary antibody in an ABC technique with 3,3′-diaminobenzidine and H2O2. (**a**–**h**) Original magnification, ×400.

Morphologically, PCNSL grow in a patternless manner in the CNS parenchyma and exhibit a marked angiocentric growth with the splitting of the blood vessel reticulin fiber network (Figure 7a,b). The tumor cells exhibit large, basophilic nuclei with one or more nucleoli, as well as a slim, eosinophilic cytoplasm. Necrosis may occur but is seen more frequently in Epstein–Barr virus (EBV)-related immunodeficiency-related lymphomas. Notably, with rare exceptions PCNSL is EBV-negative [1,2,6]. Tumor cells are characteristically intermingled with reactive CD3+, CD4+, CD8+ T, and CD20+ B cells, macrophages, activated microglia, and astrocytes [1,2].

The key pathogenetic events outlined above have a strong impact on the molecular and phenotypic characteristics of proliferatively and mitotically active malignant CD19+CD20+CD79a+ B cells (Figure 7c,d). Their late GC exit phenotype is evidenced by the expression of BCL6 and MUM1/IRF4 (Figure 7e,f) in the absence of plasma cell markers including CD38 and CD138. CD10 expression is rare and should foster search for extracerebral DLBCL, as the CD10 antigen is more frequently expressed in systemic DLBCL that may have metastasized to the CNS [70,71]. Characteristically, the tumor cells show a combined expression of BCL2 and MYC (Figure 7g,h) in the absence of translocations of these genes [76]; instead, BCL2 overexpression is associated with a gain of 18q21, which is seen in about 45% of PCNSLs [16,27], though a causal relationship between gain in 18q21 and BCL2 overexpression in non-GCB-type DLBCLs cannot yet be ultimately proven due to confounding factors [77]. Interestingly, in extracerebral DLBCL the group of BCL2 and MYC double expressors has been associated with a poor prognosis [78]. This suggests that the BCL2+MYC+ phenotype may also be prognostically relevant in PCNSL. Even more interestingly, the favorable group identified in extracerebral DLBCL that lacks both BCL2 and MYC expression was entirely absent from PCNSLs [76]. Sustained TLR, BCR, and NF-κB signaling leads to a high level of mitotic and proliferative activity (>70–90% Ki-67+ cells, Figure 7d). A lower level of proliferative/mitotic activity raises the differential diagnosis of other B cell lymphomas—e.g., low-grade B cell lymphomas, including MALT lymphoma. The inability to correctly perform CSR is reflected by the consistent IgM/IgD phenotype. Thus, the expression of other IG classes should lead to questions regarding the diagnosis of PCNSL and stimulate searches for tumors outside the CNS.

The biopsy-based characterization of the tumor immunophenotype is required for the diagnosis and classification of PCNSL. The molecular analysis of IG genes to identify a clonal B cell population with somatically mutated rearranged IG genes is dispensable in unequivocal cases but may be helpful in diagnostically difficult cases, particularly when patients have received corticosteroids prior to biopsy. Steroids support rapid tumor cell apoptosis, leaving brain tissue with large numbers of activated Ki-67+ CD3+ T cells and CD68+ macrophages and thus leading to the differential diagnosis of T cell lymphoma, inflammatory demyelination/multiple sclerosis, and other inflammatory conditions [70]. Prevailing resorption with high numbers of macrophages, activated CD3+ T cells, and thickened “empty” blood vessel walls without blasts or lymphocytes in the vessel wall should lead to the differential diagnosis of corticosteroid-mitigated PCNS-DLBCL. However, searches for a clonal population may lead to pseudoclonality due to the low numbers of non-malignant B cells. In up to 50% of cases, at least one repeat biopsy is required to establish the diagnosis of PCNSL [70,71].

In contrast to brain tissue analysis, CSF is of limited diagnostic value. Even if tumor blasts can be identified in the CSF, which may require repeated puncture, immunohistochemistry, and flow cytometry analyses, the precise classification of lymphoma is usually not possible. Thus, CSF analysis generally cannot substitute for brain biopsy.

Interestingly, CSF analysis may be a valuable tool for monitoring disease activity and the patient’s response to therapy. Recently, Grommes et al. [79] detected tumor DNA circulating in the CSF that vanished upon the implementation of effective therapy. In contrast to CSF, serum is a less suitable means for monitoring disease activity, as plasma-derived cell-free DNA was found not to harbor somatic mutations of the MYD88 and CD79B driver genes in newly diagnosed PCNSL when blood was collected prior to neurosurgery, i.e., without traumatic blood–brain barrier alterations that may allow the leakage of cell-free tumor DNA into the blood [21]. Remarkably, a low threshold level of MYD88 hotspot mutations was already found to be detectable in healthy persons, indicating that B cells might acquire these mutations even under physiological conditions [21]. In this regard, one might hypothesize that the acquisition of MYD88 mutations occurs in a similar manner to that of BCL2 mutations, suggesting the occurrence of an age-related process [80].

## 7. Conclusions and Perspectives

Intense research in the last few decades has led to the major aspects of PCNSL pathogenesis being deciphered. PCNSLs are characterized by their unique geno- and phenotype among DLBCLs, which results from the failure of B cell differentiation and the lack of appropriate control of differentiation steps. The acquisition of survival-fostering mutations together with the non-initiation of apoptosis upon the fulfillment of criteria that normally qualify the cell for elimination prior to and after the entrance of the GC establishes a vicious cycle, yielding a cell trapped in the GC with a high mutational load of IG genes, aberrant SHM of oncogenes, genomic instability, and inability to terminate the GC reaction. The ongoing GC program increases the degree to which the tumor cell fits the microenvironment of the target organ, as it continuously increases the reactivity of a plethora of CNS proteins that can be recognized by BCR, thus further fueling BCR signaling and proliferation and/or BCR-independent cell–cell interactions. Finally, the loss of MHC class I antigens adds to the escape from a potential anti-tumor T cell response. Thus, the CNS microenvironment has been found to be of particular relevance for PCNSL pathogenesis, allowing tumor cell survival at multiple levels and finally yielding a situation in which lymphoma cells and resident CNS cells fit perfectly in a lock-and-key principle.

Regarding the development of novel therapeutic strategies to improve patients’ prognosis, molecular alterations that converge on common pathways, i.e., the TLR, BCR, and NF-κB signaling pathways, are attractive targets. However, pathway activation may be the end-result of several distinct changes. Thus, in individual patients, therapeutic targets should be studied in the tumor biopsy to identify the optimal therapeutic regimen for the diseased patient. Finally, from a diagnostic point of view, restricting the diagnosis of PCNSL to the vast majority of cases showing an MCD/C5/MYD88 mutational profile should be considered. As a minimum, DLBCL with a clearly different mutational profile should be clinically and extensively staged for extra-CNS disease.

## Figures and Tables

**Table 1:** Characteristic alterations in PCNSL.

Alterations	Frequencies [%] (Number of Cases)		Techniques	References
Average mutation frequencies of variable regions of IGHV genes		13 (10)	IG PCR; Sanger sequencing	[6]
		18 (5)	IG PCR; Sanger sequencing	[11]
		10 (50)	IG PCR; Sanger sequencing	[12]
Ongoing process of SHM		100 (3/3)	IG PCR; cloning; Sanger sequencing	[6]
		60 (3/5)	IG PCR; cloning; Sanger sequencing	[11]
Aberrant SHM targeting genes with oncogenic potential		90 (9/10)	PCR; Sanger sequencing	[13]
		100 (9/9)	WES	[14]
Impaired CSR with Sµdel	IgM/IgD IgG Sµdel	100 (11/11) 0 (0/11) 64 (7/11)	IG PCR, IGHC-RT-PCR, LD-PCR; Sanger sequencing, Southern blot	[15]
Recurrent translocations affecting the *BCL6* gene		23 (3/13)	FISH; LDI-PCR; Sanger sequencing	[16,17]
		38 (14/37)	FISH; LDI-PCR; Sanger sequencing	[18]
		17 (13/75)	FISH	[19]
Recurrent translocations affecting the IG loci		38 (5/13)	FISH	[16]
		13 (10/75)	FISH	[19]
*MYD88* L265P mutations		36 (5/14)	PCR; Sanger sequencing	[20]
		85 (23/27)	NGS	[21]
		86 (12/14)	WES; RNA-Seq	[22]
*CD79B* Y196X mutations		8 (2/25)	PCR; Sanger sequencing	[23]
		59 (16/27)	NGS	[21]
		64 (9/14)	WES; RNA-Seq	[22]
Other alterations of the B cell receptor pathway	*INPP5D* *CBL* *BLNK*	20 (5/25) 4 (1/25) 4 (1/25)	PCR; Sanger sequencing	[23]
Mutations of *PRDM1*		19 (4/21)	PCR; Sanger sequencing	[24]
Mutations of *TBL1XR1*		14 (4/29)	WES; PCR; Sanger sequencing	[25]
		36 (5/14)	WES; RNA-Seq	[22]
18q21 gains (*BCL2* and *MALT1*)		38 (5/13)	FISH	[16]
		22 (2/9)	Array CGH	[26]
Chromosome 12 gains		44 (4/9)	Array CGH	[26]
		26 (5/19)	SNP array	[27]
9p13 gains (*PAX5*)		21 (4/19)	SNP array	[27]
7q31 gains		21 (4/19)	SNP array	[27]
10q23 losses (*PTEN*)		21 (4/19)	SNP array	[27]
9p21 losses (*CDKN2A*)		50 (10/20)	FD-PCR	[28]
		32 (6/19)	SNP array	[27]
		44 (16/36)	WES	[29]
8q12 losses (*TOX*)		32 (6/19)	SNP array	[27]
6p21 losses (MHC locus)		56 (5/9)	Array CGH	[26]
		74 (14/19)	SNP array	[27]
		79 (23/29)	WES; PCR; Sanger sequencing	[25]
6q21 losses (*PRDM1*)		56 (5/9)	Array CGH	[26]
		52 (10/19)	SNP array	[27]
Cell-of-origin	ABC non-cl GCB	24 43 33	RNA array	[30]
**Summary**: Germinal center (GC) B cell-derived tumor cells with a GC exit phenotype; *PIM1*, *MYD88*, and *CD79B* mutations; activated NF-kB and BCR pathways; frequently deleted *CDKN2A* and MHC loci with a high expression of *BCL2* and *MYC*, currently summarized as group MCD/C5/MYD88.				

IG: immunoglobulin; IGHV: IG heavy-chain variable region; PCR: polymerase chain reaction; SHM: somatic hypermutation; CSR: class switch recombination; Sµdel: switch µ region deletion; WES: whole-exome sequencing; RT-PCR: reverse transcriptase PCR; LD-PCR: long-distance PCR; FISH: fluorescence in situ hybridization; LDI-PCR; long-distance inverse PCR; SNP: single-nucleotide polymorphism; NGS: next-generation sequencing; RNA-Seq: whole-transcriptome (RNA) sequencing; CGH: comparative genomic hybridization; FD-PCR: fluorescent differential PCR; ABC: activated B cell-like; non-cl: non-classifiable; GCB: germinal center B cell-like.
